# Exploring the therapeutic mechanism of Baduanjin in the treatment of elderly stable angina pectoris based on the gut microbiota–lipid metabolism spectrum: Study protocol for a randomized controlled trial

**DOI:** 10.3389/fpubh.2022.1027839

**Published:** 2022-10-31

**Authors:** Xiao Jin, Shengjie Yang, Jing Lu, Yujuan Li, Yixi Zhao, Dan Li, Xinyue Wang, Longtao Liu, Min Wu

**Affiliations:** ^1^Guang'anmen Hospital, China Academy of Chinese Medical Sciences, Beijing, China; ^2^Beijing University of Chinese Medicine, Beijing, China; ^3^Department of Cardiology, Xiyuan Hospital, China Academy of Chinese Medical Sciences, Beijing, China; ^4^Department of Cardiology, Guang'anmen Hospital, China Academy of Chinese Medical Sciences, Beijing, China

**Keywords:** Baduanjin exercise, elderly, stable angina pectoris, randomized controlled trial, intestinal microflora

## Abstract

**Importance:**

Stable angina pectoris (SAP) often occurs in the elderly and is relatively stable for 1–3 months; however, if patients do not receive effective treatment, life-threatening acute myocardial infarction could occur. Patients with different clinical types of coronary heart disease have different intestinal flora. Baduanjin, a traditional Chinese Qigong, has been used as adjuvant therapy to improve the symptoms of patients with SAP.

**Objective:**

To determine the effect of Baduanjin exercise on the symptoms of patients with SAP and the intestinal flora, explore the action links and targets of Baduanjin intervention in elderly patients with SAP, and explain its mechanism.

**Design:**

A single-center, single-blind, randomized controlled trial. Patients and outcome assessors were blinded to group allocation.

**Setting:**

The trial will be conducted at Guang'anmen Hospital of China Academy of Chinese Medical Sciences.

**Participants:**

One hundred and eighty patients aged 60 to 80 years with stable angina pectoris (I–III) were intervened for 8 weeks and followed up for half a year.

**Interventions:**

Among the screened patients, 180 patients will be randomly assigned to either the Baduanjin or the control group at a 1:1 ratio (exercise duration: for 3–5 times a week, for 8 weeks) of moderate-intensity Baduanjin or free activities.

**Main and secondary results:**

The main result is the total effective rate for angina pectoris symptoms; secondary results include the duration of angina pectoris, number of angina pectoris episodes per week, nitroglycerin consumption, nitroglycerin reduction rate, Seattle angina score (SAQ), quality of life (SF-36),Traditional Chinese Medicine (TCM) syndrome scores, electrocardiogram (ECG) changes, blood lipid serum hypersensitive C-reactive protein levels, intestinal flora changes, serum changes in the intestinal flora metabolite Trimetlylamine oxide (TMAO), and non-targeted liposome detection. Adverse events will be recorded throughout the experiment, and the data will be analyzed by researchers who did not know about the assignment.

**Discussion:**

This study provides compelling evidence for at-home use of Baduanjin exercise to relieve SAP-associated symptoms.

**Trial registration:**

This study was approved by the ethics committee of Guang'anmen Hospital of China Academy of Chinese Medical Sciences (2022-121-KY). The trial has been registered in Chinese Clinical Trial Registration Center (ChiCTR2200062450).

## Introduction

Angina pectoris is a common cardiovascular disease seen in outpatient departments and is characterized by insufficient blood supply caused by coronary atherosclerosis, resulting in myocardial hypoxia and ischemia, followed by paroxysmal chest pain (1), precordial discomfort, and other symptoms, especially in the elderly (2). Chronic stable angina pectoris (SAP) is the most common subtype, and its clinical manifestations remain stable for ~1–3 months; however, without timely and effective diagnosis and treatment, it can easily develop into life-threatening acute myocardial infarction. Due to the symptoms caused by angina pectoris (e.g., chest pain), many elderly people restrict their daily activities, thereby leading to a relatively poor quality of life (1).

SAP was initially treated using percutaneous coronary intervention (PCI) (2). Annually, more than 500,000 patients with SAP undergo PCI. Clinical results from revascularization and active drug evaluation trials showed little difference in the incidence and mortality of myocardial infarction between patients with stable coronary heart disease who received coronary intervention and the control group (3). The benefits of coronary revascularization are limited to improving quality of life and cannot decrease the rate of cardiovascular events (4). Emphasis should be placed on optimizing lifestyle factors and preventative therapy, such as intake of lipid-lowering and antiplatelet drugs, to reduce the risk of cardiovascular events and death. Anti-angina drugs, such as β-receptor blockers, nitrates, or calcium channel blockers, should be started to improve the symptoms of angina pectoris (5). Phase I of a cardiac Rehabilitation (CR) program is recommended by the American Heart Association and the European Society of Cardiology as part of the comprehensive care for patients with coronary heart disease. In particular, exercise therapy has always been regarded as a core element (6–9). The results of a recent systematic analysis also support this view (10). However, the proportion of people over 60 years old participating in traditional CR is low (6, 11). Due to poor health and environmental barriers, most older people cannot meet the recommendations of the American Heart Association on physical activity for older people (12, 13). In the United States, <40% of elderly patients with coronary heart disease participate in CR (6).

Baduanjin is the most widespread ancient Chinese technique as it is a moderate-intensity-free activity, can be easily practiced, and is not restricted by venue (14, 15). Baduanjin comprises gentle and slow movements, combined with deep breathing and relaxation. Long-term exercise can strengthen health, bright ears and eyes, and prolong life. As a health-preserving method, Baduanjin has a 1,000-year history. It contains eight easy-to-learn movements and has a positive effect on all organs of the human body (16). Baduanjin can delay left ventricular remodeling in patients with myocardial infarction by improving energy metabolism and inhibiting inflammation (17). Additionally, it has a positive role in protecting vascular endothelial function, alleviating dyslipidemia, stabilizing blood sugar and blood pressure, and improving quality of life (18–22). Furthermore, short-term endurance exercise contributes to improving the richness of intestinal microflora (23).

Based on this background, we designed a single-center, single-blind, randomized controlled trial (RCT) to compare the efficacy and safety of 8 week Baduanjin exercise and moderate-intensity aerobic walking in elderly patients with SAP. We also assessed nitroglycerin reduction dose, angina pectoris frequency, Seattle angina pectoris score (SAQ), quality of life (SF-36), TCM syndrome score, ECG changes, blood lipids, serum hypersensitive C-reactive protein and intestinal flora changes.

## Materials and methods

### Design

**Figure 1:**
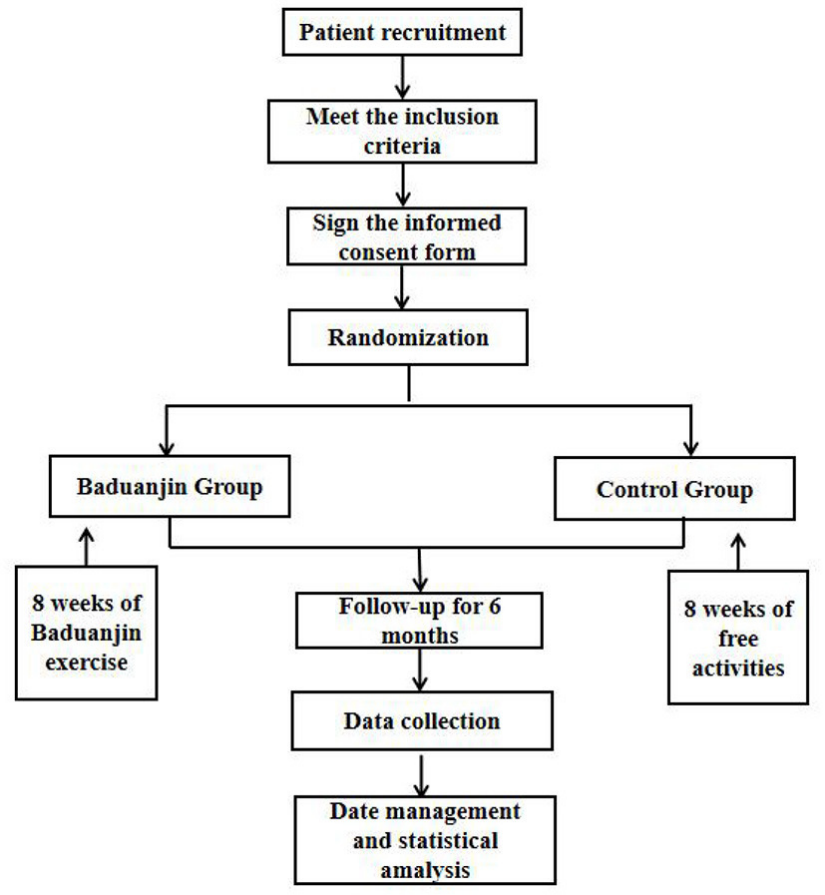
Flowchart of the clinical trial design.

This single-center, randomized controlled trial will be conducted in Guang'anmen Hospital of China Academy of Chinese Medical Sciences. After providing written informed consent, 180 participants will be randomly assigned to the medium-intensity Baduanjin or medium-intensity control groups (free activities). Medium-intensity is defined as energy consumption (calories) per unit time, which is 3 to 6 times higher than that during meditation. The intervention time will be 8 weeks, followed by 6 months of follow-up, and the results will be evaluated immediately at the end of the follow-up period. Figure 1 illustrates the flow of this experiment; the scheme of the clinical trial is in line with the standard program project: intervention trial guidelines recommend (24) and follow the comprehensive standards for reporting trials (25).

### Recruitment

Through a series of recruitment strategies such as putting up posters in the hospital, patients will be recruited from the Department of Cardiology and Comprehensive Department of Guang'anmen Hospital. Researchers will communicate with potential patients and provide relevant information relating to the study, including the purpose, process, interventions, and potential adverse reactions. Researchers will screen the patients, and those who meet the inclusion criteria and volunteer to participate in the study will provide written informed consent.

### Study population

The diagnostic criteria for chronic SAP refers to the 2019 ESC guidelines for the diagnosis and management of chronic coronary syndromes (26).

#### Inclusion criteria

The inclusion criteria are as follows:

(1) Meet the SAP diagnostic criteria,
(2) Age between 60 and 80 years old,
(3) Classified as I–III based on severity, according to the Canadian Cardiovascular Society (CSS) grading of angina pectoris,
(4) Sign the informed consent form.

#### Exclusion criteria

The exclusion criteria are the following:

(1) Severe arrhythmia and moderate cardiac insufficiency (EF <40%);
(2) Uncontrolled hypertension, systolic blood pressure (SBP) ≥180 mmHg, and diastolic blood pressure (DBP) ≥100 mmHg;
(3) Three-month recent history of AMI or PCI, coronary artery bypass grafting, severe trauma, and cerebrovascular accident;
(4) Severe shock and primary organ diseases (such as of the liver, kidney, lung, etc.);
(5) Obvious infection, fever, and severe anemia;
(6) Severe joint disease;
(7) Antibiotic use or participation in any antibiotic, probiotic, or laxative research in the 3 months preceding the study;
(8) Gastrointestinal diseases such as diarrhea and ulcerative colitis in the past 2 months.

#### Criteria for withdrawal and removal

(1) Researchers will further exclude the following patients:
  ① Those who were not suitable to continue with the trial;
  ② Those with poor compliance, which affects their judgment of efficacy and safety;
  ③ Those with disease progression to acute coronary syndrome;
  ④ Those unable to perform Baduanjin exercises due to serious musculoskeletal problems.
(2) Self-withdrawal is characterized as follows:
  ① Unwillingness or inability to continue with the clinical trial for any reason;
  ② Not explicitly intending to withdraw from the trial but no longer being treated and tested.

### Randomization and blinding

Participants will be randomly assigned to the Baduanjin or control groups with a proportion of 1:1, using a random number grouping table generated according to the number of assigned cases and random proportion. The random number table will be generated by using SAS 9.0, emergency letter preparation will be completed by people not involved with this clinical trial, and grouping information will be kept in a sealed, opaque envelope. The order of recruitment of participants is natural, and after enrollment, the researchers will open the envelopes in turn to determine the patients' grouping information.

In view of the different forms of intervention, blinding of the researchers, participants, and coaches throughout the experiment is not feasible. However, the laboratory technicians and researchers conducting the statistical analysis will remain blinded.

### Intervention

#### Baduanjin group

**Figure 2:**
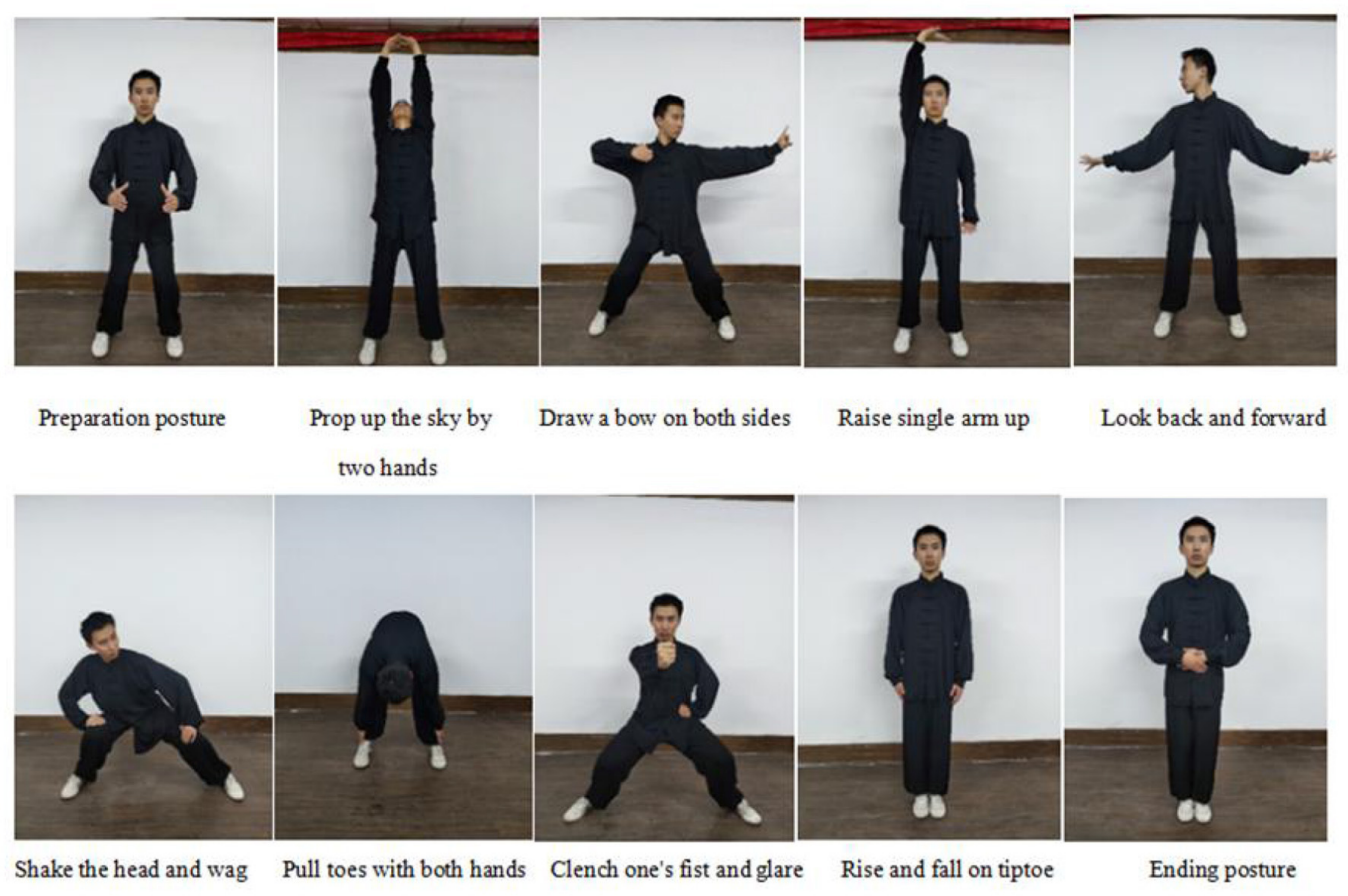
Ten postures of Baduanjin exercise.

The Baduanjin group participants will focus on practicing at the Guang'anmen Hospital. During hospitalization, researchers guided and supervised the exercise, and after discharge, special researchers will supervise telephonically or through WeChat. Based on basic Western medicine treatment and health education, patients will perform eight stages of brocade exercise. Each exercise lasts about 30 min, including 5 min of warm-up exercise, 20 min of Baduanjin exercise, and 5 min of relaxation activities, lasting for 8 weeks, 3–5 times a week. The training program was formulated according to the “Baduanjin of Fitness Qigong,” issued by the State Administration of Sports in 2003, with a total of 10 postures (Figure 2). Participants should not be exercising extra.

#### Control group

The control group will perform exercises based on basic western medicine treatment and health education. During hospitalization, we will conduct supervisory training for the control group to ensure that patients exercise 3–5 times a week (such as walking). After discharge, there will also be special researchers to supervise through Wechat or the phone. The main form of exercise is moderate-intensity free exercise, lasting 8 weeks, for 3–5 times a week.

#### Concomitant treatment

All patients will receive routine treatment, such as aspirin, clopidogrel, statins, ß-receptor blocker, and angiotensin-converting enzyme inhibitors. Drug changes of all patients will be recorded in the comprehensive drug record sheet.

### Sample size

The sample size was calculated according to the effective rate of the Baduanjin group and the control group. Referring to the previous research (27), it is assumed that the effective rate of the combination of traditional Chinese medicine and western medicine is 76.2%, and the effective rate of western medicine is 45.8%. Based on the class I error rate of α = 0.05 and the efficacy of 90% (II error rate of β = 0.1), bilateral test, and considering the shedding of 20%, the experiment was expanded to 69 people. Because the reference study is drug intervention, considering that this study is non-drug intervention, we continue to expand the sample size to 90 people in each group, a total of 180 people.


$$\begin{array}{l} n=\frac{{\left(u_{\alpha }+u_{\beta }\right)}^{2}\left(1+1/k\right)p\left(1-p\right)}{{\left(p_{e-}p_{c}\right)}^{2}} \end{array}$$

### Outcome measurements

Table 1 provides a detailed overview of the items that will be measured for the primary and secondary results, and the time of data collection. The main outcome indicators will be measured prior to the experiment (week 0), after the end of the trial (week 8), and at 3 and 6 months after enrollment. Data on the secondary outcome indicators, including the duration of angina pectoris, number of angina pectoris weeks, nitroglycerin consumption, nitroglycerin reduction rate, SAQ, quality of life (SF-36), and TCM syndrome scores, will be collected at weeks 0, 4, and 8 and months 3 and 6. Details on secondary outcome indicators, including electrocardiogram (ECG) readings, blood lipid and hs-CRP levels, and intestinal microflora, will be collected during weeks 0 and 8. All measured outcomes will be recorded in the case report form.

**Table 1:** Schedule of trial measurements for primary and secondary outcomes.

**Study phase**		**Screen/enroll**	**Allocation**	**Intervention period**	**Intervention end**	**Follow-up period**	**Follow-up period**
**Time points**		**Week 0**		**Week 4**		**Month 3**	**Month 6**
Screening/enrollment	Eligibility screening	•	•	—	—	—	—
	Informed consent	•	—	—	—	—	—
	Baseline measurement	•	—	—	—	—	—
	Random allocation	—	•	—	—	—	—
Intervention	Baduanjin exercise	—	•				•
	Free activities	—	•				•
Outcome assessment	Total effective rate	—	•	•	•	•	•
	Duration	—	•	•	•	•	•
	Attack frequency	—	•	•	•	•	•
	Dosage	—	•	•	•	•	•
	Stopping rate of nitroglycerin	—	•	•	•	•	•
	SAQ	—	•	•	•	•	•
	SF-36	—	•	•	•	•	•
	ECG	—	•	—	•	—	—
	Serum lipids	—	•	—	•	—	—
	Hs-CRP	—	•	—	•	—	—
	The TCM syndrome scores	—	•	•	•	•	•
	Composition of gut microbiota	—	•	—	•	—	—
	TMAO	—	•	—	•	—	—
Safety index	—	—	•	—	•	—	—
Adverse events	—	—	•				•

#### Primary outcome

The main outcome index is the total effective rate of angina pectoris symptoms, described in detail in Tables 2, 3. Data will be collected at baseline, weeks 4 and 8, and months 3 and 6 after enrollment.

**Table 2:** Angina pectoris symptom scoring standard.

**Angina pectoris symptom scoring standard**			
**Number of seizures**		**Symptom**	**Integral**
	None	No attack within a week	0 point
	Mild	Attack 2–6 times a week	2 point
	Moderate	Attack 1–3 times a week	4 point
	Severe	Attacks more than 4 times a week	6 point
The degree of pain	None	No attack within a week	0 point
	Mild	Heavier than daily activities of physical activity caused angina pectoris	2 point
	Moderate	Daily physical activities cause angina pectoris, and daily activities are restricted.	4 point
	Severe	Physical activities that are lighter than daily activities cause angina pectoris, and daily activities are obviously limited.	6 point
The duration of the pain	None	No attack within a week	0 point
	Mild	The pain lasts ≤5 min	2 point
	Moderate	5 min < The pain lasts <10 min	4 point
	Severe	The pain lasts ≥10 min	6 point
Dosage of nitroglycerin	None	None	0 point
	Mild	Take 1–4 tablets a week	2 point
	Moderate	Take 5–9 tablets a week	4 point
	Severe	Take more than 10 tablets a week	6 point
		Total	____point

**Table 3:** Evaluation of curative effect on angina pectoris symptoms.

**Evaluation of curative effect on angina pectoris symptoms**	
Significant effect	Disappearance of symptoms or almost disappearance of angina pectoris symptom efficacy index ≥70%.
Effective	The frequency, degree and duration of pain attacks were significantly reduced by 30% ≤ angina pectoris symptom index <70%.
Invalid	The symptoms are the same as those before treatment. 0 ≤angina pectoris symptom efficacy index <30%.
Aggravate	The frequency, degree and duration of pain attacks were aggravated, the score of angina pectoris was increased, and the curative effect index of angina pectoris symptoms was <0.

Description: angina symptom efficacy index = (pre-trial angina symptom score-post-trial angina symptom score)/pre-trial angina symptom score × 100%. The symptom score of angina pectoris is the sum of the scores in Table 2. Total effective rate = (significant effect + effective)/sample size × 100%.

#### Secondary outcome

(1) Clinical symptoms: after confirming that the patients meet the inclusion criteria, researchers will ask about and record the clinical symptoms of the patients (including the duration of angina pectoris, frequency of weekly attacks, and the dosage of nitroglycerin) at baseline, weeks 4 and 8, and months 3 and 6 after enrollment.
(2) Stopping rate of nitroglycerin: This will be recorded at the baseline and at weeks 4 and 8 and months 3 and 6 after enrollment.
  ① Significant effect: discontinuation of nitroglycerin after treatment;
  ② Effective: the dosage of nitroglycerin after treatment is >50% less than that before treatment;
  ③ Ineffective: after treatment, the dosage of nitroglycerin decreased by <50% or increased, compared with that before treatment.
(3) SAQ: This is a 19-item self-administered questionnaire measuring five dimensions of coronary artery disease: physical limitation (question 1), anginal stability (question 2), anginal frequency (question 3–4), treatment satisfaction (question 5–8), and disease perception (question 9–11) (28). The 19 items will be scored individually to obtain the total SAQ score, which is transformed into a standard score according to this formula: Standard score = (actual score – lowest score of the dimension) x100/(highest score of the dimension – lowest score of the dimension). Patients' quality of life and body function are proportional to the score, which will be recorded at the baseline and weeks 4 and 8 and months 3 and 6 after enrollment.
(4) SF-36: The MOS item short will be used from the health survey (SF-36) to assess patients' quality of life. As a concise health questionnaire, SF-36 comprehensively summarizes the quality of life of the subjects from eight aspects: Physical Functioning (PF), Role-Physical (PR), Bodily Pain (BP), General Health (GH), Vitality (VT), Social Functioning (SF), Role-Emotional (RE), and Mental Health (MH). There are 36 questions, and the quality of life is proportional to the score (29). SF-36 is effective in detecting the health perception of the general population, easy to use, and highly accepted by patients, and studies have proven it to be a promising tool due to its high reliability (30, 31). SF-36 will be recorded at baseline, weeks 4 and 8, and months 3 and 6.

$$\begin{array}{l} \begin{array}{c} \text{PF}=(\text{Actual score}-\;20)/40\times 100;\\ \text{PR}=(\text{Actual score }-4)/4/4\times 100\\ \beta \text{P}=(\text{Actual score}-2)/10\times 100;\\ \text{GH}=(\text{Actual score}-5)/20\times 100\\ \text{VT}=(\text{Actual score}-4)/20\times 100;\\ \text{SF}=(\text{Actual score}-2)/8\times 100\\ \text{R}\epsilon =(\text{Actual score}-3)/3\times 100;\\ \text{MH}=(\text{Actual score}-5)/25\times 100 \end{array} \end{array}$$

(5) ECG: This will be recorded at baseline and after the intervention. Routine 12-lead ECG will performed with the patient at rest. The evaluation of the standard curative effect of ECG is based on the curative effect standard of coronary heart disease and angina pectoris (32) (Table 4).
(6) Serum lipids: Venous blood samples (5 mL) will be collected before and 8 weeks after intervention. After coagulation, samples will undergo centrifugation at 3,000 r/min. The supernatant will be collected after 15 min and serum lipid levels (triglycerides, cholesterol, low-density lipoprotein cholesterol, high-density lipoprotein cholesterol, lipoprotein-a, small, dense low-density lipoprotein cholesterol) will be determined using enzyme-linked immunosorbent assay (ELISA).
(7) Hs-CRP: Venous blood samples (5 mL) will be collected before and 8 weeks after intervention. After coagulation, the serum will be centrifuged at 2,000 r/min for 20 min, and the CRP level will detected quantitatively using ELISA.
(8) The TCM syndrome scores of patients will be recorded at the baseline and at weeks 4 and 8 and months 3 and 6 after enrollment. TCM syndrome score indexes for patient evaluation includes chest pain, chest tightness and shortness of breath, palpitation, and fatigue. Each index is allotted 1–3 points, and the total score is 12 points. A higher TCM score suggests a more serious condition (33).
(9) Intestinal flora: The feces were collected by the patients at home (at the baseline and the eighth week), the researchers distributed a sterile collection tube containing the preservation solution and a sterile spoon to the patients, and explained in detail to the patients how to collect the feces. After defecation, the patient uses a sterile spoon to collect from the inside of the stool and put it in the collection tube. Each patient keeps two specimens, each of which is about 1 g. The researchers then recycled the feces at −80°C for storage. High-throughput 16SrRNA sequencing will be used to effectively identify microbial species (including eight phyla: *Firmicutes, Bacteroidetes, Verrucomicrobia, Spirochaetae, Proteobacteria, Cyanobacteria, Saccharibacteria*, and *Synergistetes*) and their abundance in a particular environment. The significant differences in intestinal microecological composition and distribution abundance will be compared between the two groups, before and after treatment. The metabolic genes related to intestinal flora will be detected using macrogenomic sequencing, and the differences in KEGG metabolic pathway and species function will be predicted and analyzed.
(10) Metabolomics: Fasting blood samples will be drawn at baseline and at the eighth week, and the fresh whole blood samples will be immediately sent to the laboratory under cold storage (ice-box transfer) and centrifuged at 4°C, at 3,000 r/min for 10 min. Finally, the supernatant (plasma) will be frozen at −80°C. The TMAO content will be determined using high-performance liquid chromatography–tandem mass spectrometry (HPLC-MS), and a standard curve will be drawn to calculate the serum TMAO concentration in patients with SAP. Non-targeted liposome detection, data preprocessing and database search, differential metabolite analysis, and bioinformatics analyses will be conducted using HPLC-MS.

**Table 4:** Efficacy criteria of electrocardiogram.

**Efficacy criteria of electrocardiogram**	
Significant effect	ECG returns to roughly normal or normal range of electrocardiogram.
Effective	After treatment, the ST segment rises ≥0.05 mv, but does not reach the normal level, the main lead causes the T waves to become shallow >25%, or the T wave changes from flat to upright; severe arrhythmia is improved.
Invalid	ECG is roughly the same as before.
Aggravation	The ST segment decreased more than 0.05 mV after treatment. In the main ECG leads, T wave changes more deeply (up to 25%); or T wave changes from upright to flat, or upright T wave inversion, ectopic rhythm, atrioventricular or ventricular block.

#### Safety index

Safety indices will be routinely assessed using blood, urine, and stool samples and by performing liver and renal function tests. These will be measured at baseline and the eighth week after enrollment.

### Data collection and management

If participants deviate from the intervention program, additional participants will be included to minimize data loss during the study period. Patients will be supervised and guided by specialized researchers throughout the intervention. At the fourth and eighth weeks, participants will be required to return to the hospital for scale (Such as clinical symptoms, SAQ and TCM syndrome scores) or clinical-related tests, and regular telephonic follow-up will be conducted by researchers during the follow-up period. Researchers remind patients to undergo daily moderate-intensity training, and those who deviate from the intervention plan will receive telephonic reminders, to ensure the integrity of the study data.

Basic patient information and relevant information required for the study will be recorded in the printed case report form (p-CRF). The patient's medical records (medical records, test sheets, etc.) will be stored in the hospital, and the doctor records the patient's test results in the observation medical records. Only authorized researchers, ethics committee members, and relevant research management department members will be able to access patient records following reasonable request. The name and identity of the patient will not appear in the study and published papers. The research team will do everything possible to protect the privacy of patients' personal medical data from disclosure within the scope permitted by law.

### Quality control

Quality control will be conducted throughout the test. Before the start of the trial, all researchers will receive unified clinical trial training to fully understand the trial scheme, trial process, and shared tasks. For example, the Independent Data and Safety Monitoring Committee (DSMB), composed of two cardiologists and a statistician, will closely monitor the participants' trial data; to prevent data loss, researchers will be responsible for reminding participants to complete the exercise. In addition, investigators will receive unified training, including in regard to data management and filling out of the p-CRF to ensure high-quality completion of the trial.

### Statistical analysis

A full-analysis set (FAS), per-protocol analysis set (PPS), and safety analysis set (SAS) will be created according to the principle of intentional therapy (34). FAS participants are identified as those who received at least one exercise. Those who adhere to the treatment plan but changed groups midway are part of the PPS. For safety indicator assessment, researchers will use the SAS to analyze the incidence of adverse events and laboratory indicators. Statistical analysis will be conducted by third-party statisticians blinded to experimental grouping and intervention methods, using SPSS 25. We will analyze the baseline demographic data to determine the comparability between the two groups. Mean ± standard deviation will be used for continuous variables that conform to normal distribution, and median and quartile methods will be used for continuous variables that do not conform to normal distribution. The Chi-square test or Fisher's exact test will be used to compare classified counting data. The paired *t*-test will be used to compare differences within the group, and the independent-sample *t*-test will be used to compare differences between groups. If the data do not conform to normal distribution, a Wilcoxon paired test and Mann-Whitney U test will be used to analyze intra- or inter-group differences. *P* < 0.05 is considered as statistically significant.

### Safety assessment

The safety index is used to evaluate the security of this study. If adverse events (such as disability, death, etc.) occur during the study, researchers will assess the relationship between the event and intervention and report it to the Ethics Committee and DSMB to determine whether those participants should withdraw from the trial. During the study period, any study-related damage will be treated accordingly, following identification by the Beijing Medical Accident Identification Committee, and followed up until recovery or until the patient became stabilized, with the expenses covered by Guang'anmen Hospital.

## Discussion

This experiment will evaluate the efficacy and safety of Baduanjin in patients with SAP and detect changes in intestinal flora and the metabolite TMAO.

Angina pectoris is a cardiovascular disease characterized by chest pain. Clinically, it is divided into SAP and unstable angina pectoris (35). SAP is the most common symptom of coronary heart disease. Although the overall prognosis is good, the annual incidence of events is 1–2% (36). For many people, its symptoms seriously affect their lifestyle. In severe cases, it can develop into myocardial infarction. As a traditional Chinese Qigong, Baduanjin has been used in health care for a long time. Its movements are simple and unrestricted by location (15). Studies have shown that Baduanjin can effectively improve cardiac function (17).

Additionally, the occurrence of SAP is reportedly related to changes in intestinal microflora (37). Exercise can increase the concentration of short-chain fatty acids in feces and the ability of intestinal microflora to produce short-chain fatty acids (38). Therefore, we hypothesize that Baduanjin may be related to the function of intestinal microflora. Further, we explored the mechanism of its effect on SAP based on the composition of intestinal microflora. If successful, the trial will provide SAP patients with a good exercise program and encourage them to improve their quality of life by using Baduanjin as an adjuvant treatment.

Our experiment had several advantages: we used a strict randomized control method, which was strictly confidential to evaluators and statistical analysts, thus enhancing the reliability of the experiment; Baduanjin, as a traditional Chinese method, is a trustworthy method (having been around for years) and is easy to perform, thus increasing compliance; and Baduanjin is simple and gentle and is very easy for patients with SAP to perform. In addition, if the experiment proves to be effective, Baduanjin not only improves the quality of life of patients, but also reduces medical expenses and saves medical resources.

There were two main limitations in our experiment: it was a single-center experiment; therefore, it is smaller than multi-center experiments. It is not clear whether the Baduanjin exercise works in other patient ethnicities. In addition, it is not clear if the participants will receive additional training on this technique as it requires constant supervision and visits by researchers.

In conclusion, this experiment will confirm whether Baduanjin can be used as an adjuvant therapy for SAP. This randomized controlled trial will help to provide evidence to verify whether the mechanism of Baduanjin improving SAP is related to gut microflora.

## Trial status

Recruitment started while the manuscript was being finished.

## Ethics statement

The study was conducted in accordance with the principles outlined in the Helsinki Declaration. The research project was approved by the Medical Ethics Committee of Guang'anmen Hospital of China Academy of Chinese Medical Sciences (approval number: 2022-121-KY) on 24/06/2022. This study has been registered in the Chinese Clinical Trials Registry (ChiCTR2200062450). The specific approval of the training site was approved by Guang'anmen Hospital of the Chinese Academy of Traditional Chinese Medicine.

## Author contributions

XJ, YZ, SY, JL, YL, XW, DL, and MW developed and conceived the study. XJ, LL, and MW designed the study protocol and developed the intervention. XJ wrote the first draft of the manuscript, participated in the coordination, and implementation of the study. MW revised, finalized the study protocol, charge of coordination, and direct implementation. All authors substantially contributed to drafting the manuscript and approved the final version of the manuscript.

## Funding

This study was supported by the National Natural Science Foundation of China (http://www.nsfc.gov.cn, under Grant Nos. 81202805, 81973689, 81573821, and 82074254), the Beijing Natural Science Foundation (Nos. 7172185 and 7202176), the Special Project of Business Construction and Scientific Research of the National Clinical Research Base of Traditional Chinese Medicine of the National Administration of Traditional Chinese Medicine (No. JDZX2015262), and Science and Technology Innovation Project of China Academy of Chinese Medical Sciences (No. C12021A01413).

## Conflict of interest

The authors declare that the research was conducted in the absence of any commercial or financial relationships that could be construed as a potential conflict of interest.

## Publisher's note

All claims expressed in this article are solely those of the authors and do not necessarily represent those of their affiliated organizations, or those of the publisher, the editors and the reviewers. Any product that may be evaluated in this article, or claim that may be made by its manufacturer, is not guaranteed or endorsed by the publisher.
